# Cell Shape and Cardiosphere Differentiation: A Revelation by Proteomic Profiling

**DOI:** 10.1155/2013/730874

**Published:** 2013-09-01

**Authors:** Nanako Kawaguchi, Mitsuyo Machida, Kota Hatta, Toshio Nakanishi, Yohtaroh Takagaki

**Affiliations:** ^1^Department of Pediatric Cardiology, Tokyo Women's Medical University, 8-2, Kawada-cho, Shinjuku, Tokyo 162-8666, Japan; ^2^Department of Legal Medicine, School of Medicine, Tokyo Women's Medical University, 8-1 Kawada-cho, Shinjuku, Tokyo 162-8666, Japan; ^3^University of Toronto and University Health Network, Toronto, ON, Canada M5G1L7; ^4^Nihon Pharmaceutical University, 10281 Inacho-komuro, Kita-adachi-gun, Saitama-ken, Saitama 362-0806, Japan

## Abstract

Stem cells (embryonic stem cells, somatic stem cells such as neural stem cells, and cardiac stem cells) and cancer cells are known to aggregate and form spheroid structures. This behavior is common in undifferentiated cells and may be necessary for adapting to certain conditions such as low-oxygen levels or to maintain undifferentiated status in microenvironments including stem cell niches. In order to decipher the meaning of this spheroid structure, we established a cardiosphere clone (CSC-21E) derived from the rat heart which can switch its morphology between spheroid and nonspheroid. Two forms, floating cardiospheres and dish-attached flat cells, could be switched reversibly by changing the cell culture condition. We performed differential proteome analysis studies and obtained protein profiles distinct between spherical forms and flat cells. From protein profiling analysis, we found upregulation of glycolytic enzymes in spheroids with some stress proteins switched in expression levels between these two forms. Evidence has been accumulating that certain chaperone/stress proteins are upregulated in concert with cellular changes including proliferation and differentiation. We would like to discuss the possible mechanism of how these aggregates affect cell differentiation and/or other cellular functions.

## 1. Introduction

Two epoch accomplishments in the first decade of 21st century are changing the scope of biomedical research. The first was the completion of the human genome project [1], which enabled the onset of “Omics” or the integrative approach (System Biology) [2]. The second was the discovery of adult stem cells in human [3] followed by induction of pluripotency by Yamanaka factors (Oct3/4, Sox, Klf4, and c-Myc) in both mouse and human somatic cells [4, 5]. Adult stem cells are undifferentiated cells found throughout the body after development. They have the ability to self-renew indefinitely and have the developmental potential to generate many other cell types due to cell fate switching induced by extracellular environmental signals [3]. Plasticity of stem cells as well as the induction and reprogramming of somatic cells ignited the hope of discovering cellular therapy for the regeneration of damaged body parts. The revelation of the involvement of extracellular factors in switching cell types resulted in paradigm shift from “genetic determinism”, the paradigm that all biological processes follow the one-way instruction stored in genomes to an “environment-genome interaction” understanding.

Studies on the regulatory molecular mechanisms underlying these changes often rely on gene expression analyses with transcription profiling (transcriptome) and microarrays. These mRNA analyses, however, have limitations because of variability in mRNA stability, the translational rates of genes, and protein degradation. Indeed, several investigations revealed that some of the protein expression levels are poorly correlated with the respective mRNA levels [6, 7]. On the other hand, proteome analysis can cover a wide range of expressed proteins including unknown products and also has the potential to provide information on posttranslational modification and subcellular localization of proteins. Recent reviews of proteome analyses of embryonic stem cells show that some proteins can be used as common indicators of “stemness” [8–10]. Although proteome profiling requires more technical refinements to be readily applicable for general integrative research, the results obtained are already becoming uniquely valuable in gaining insights into a variety of the developmental processes.

The adult mammalian heart has been traditionally regarded as a terminally differentiated organ. Recent evidence, however, indicates that it has resident stem cells with self-renewing capacity. From rodent and human adult hearts, cells expressing c-kit, Sca-1, and MDR1 antigens were isolated, and they were demonstrated to be clonogenic and multipotent, with the capacity of generating cardiac myocytes, smooth muscle, and endothelial cells [11, 12]. These results indicate that cardiac stem cells reside in the heart, maintain their stem cell properties, and are capable of responding to stimuli to generate cells for repairing damaged tissue, such as in the case of heart failure or myocardial infarction [12–14]. We also isolated cardiac stem cells from the rat heart and found that they are responsive to environmental factors and are plastic cells with multilineage potential [15–19]. Out of bulk culture of these cardiac stem cells, we isolated a unique cardiosphere clone which changes its shape from round spherical cells in aggregates to flat, adherent cells [20]. The shape change was a reversible process manipulated by switching the culture condition. The comparative proteome analysis of the two cell shapes showed dramatic alteration in protein profiles, especially of metabolic alteration and the switches in the expression of chaperons members. Apart from growth factors and nutrients, we would like to address the possibility of mechanical stress applied to the cell surface as being a trigger in cell fate determination [21–25]. The concept that different extracellular environments command the intracellular activity needs to be extensively explored for manipulating stem cells for regenerative therapy. 

## 2. Isolation of Cardiac Stem Cells from the Heart

In 2002, Hierlihy et al. reported that the adult mouse myocardium retains an endogenous stem cell-like population that is activated during growth challenge [26]. In 2003, Oh et al. isolated stem cell antigen-1-positive (Sca-1^+^) cells from the adult mouse myocardium, which migrate to the injured myocardium when administered intravenously after ischemia/reperfusion [27]. Beltrami et al. reported the presence of cardiac resident stem cells by isolating c-kit^pos^ cells from adult rat hearts by using immunomagnetic beads and FACS sorting [12]. These cardiac c-kit^pos^ cells are described as self-renewing clonogenic multipotent cells that give rise to myocytes, smooth muscle cells, and endothelial cells. Messina et al. were the first to isolate similar clonogenic cardiac stem cells from human biopsy tissues [11]. Matsuura et al. used an immunopurification system to isolate Sca-1^+^ cells from the adult murine heart, which differentiated into beating cardiomyocytes after induction of differentiation by oxytocin. Sca-1^+^ cells were also induced to differentiate into cells with osteocyte or adipocyte characteristics indicating their multipotency [28]. Linke et al. used dog hearts and immunopurification and isolated c-kit^pos^, MDR1^+^, and Sca-1^+^ cells that were self-renewing, clonogenic, and multipotent [29]. These results suggest that there are multiple types of cardiac adult stem cells. 

In their study, Linke et al. minced tissues and cultured explants for 1–3 weeks. A layer of fibroblast-like cells was generated from the adherent explants, over which small phase-bright cells migrated and clustered together forming spheroids or cardiospheres (CSs) [29]. These CSs were periodically harvested by treatment with EDTA-trypsin and allowed to grow on poly-D-lysine-coated culture surfaces in specific culture media. The explant CSs were also obtained from murine hearts. Both human and murine explant CSs were self-renewing and clonogenic and reported to produce spontaneously beating cells [11]. Some of the cloned cells generated self-adherent cell aggregates or spheroids. Cardiospheres have been used for isolation of cardiomyocyte progenitor cells [11, 13]. They are a heterogeneous cell population, and, in bulk, they express several stemness genes, such as c-kit and MDR1, but also express cardiac-specific gene such as Nkx2.5 [30]. Because of their heterogeneity, some suggested that cardiomyocytes and/or cardiomyocyte progenitors were contaminants in these spheres and became beating cells when matured [31]. On the other hand, cardiospheres have been tried numerous times for the regeneration of the ischemic heart, and positive results have been reported [32–42]. Moreover, the human cardiospheres were applied to infarcted rodent hearts, and positive effects have been observed in terms of their expansion and differentiation resulting in thickening of tissue [34]. Makkar et al. reported that cardiosphere-derived cells were used for phase 1 clinical trials, and they warrant a phase 2 study [42]. Readers can refer to review articles for details about other types of cardiac stem cells [21, 22, 43–48].

## 3. Cardiospheres

Spheroids have the morphology typically observed in the early developmental stages of various cell lineages such as embryonic stem cells (ESCs), neurospheres, embryoid bodies (EBs) observed in teratocarcinoma cultures, and induced pluripotent stem cells (iPSCs) [4, 5, 49, 50]. Some tumor cells can be grown as multicellular spheroids [51]. Spheroids have a three-dimensional (3D) architecture, a core consisting of aggregated cells adhering to each other, and surface monolayer of cells surrounding the core cells. The core cells are likely to have less access to nutrients, oxygen, and growth factors because the surface monolayer of cells shields them from the environment and because they are tightly packed. In glioma spheroids, Khaitan et al. showed that average glucose consumption and lactate production are 2-3 times higher in viable spheroid cells compared to monolayer cells [52]. Since hypoxia inducible factor-1*α* (HIF-1*α*) is expressed only in spheroids, the increase in glycolysis is likely induced by hypoxia with reduced mitochondrial mass and activity. Tumor spheroids are resistant to drugs and radiation [53]. Because of this property, spheroids are likely to be more beneficial for the preservation of stem cells within these structures as hypoxic conditions mean less oxidative damage. The cell shapes inside the spheroids might also alter cellular properties, resulting in different mechanosensitivities and responses to stimuli. Armstrong et al. [54] cultured fetal chick myocardium and demonstrated that reaggregated cardiomyocytes from tissue spheroids were responsive to several growth factors whereas the cardiomyocytes maintained in monolayered, flattened cells lost this responsiveness, thus indicating that cell shape altered the internal property of cardiomyocytes [54]. 

For human studies, explant cardiosphere-derived cells (CDCs) are favored because of the advantage of being able to isolate cells from very small fragments of biopsy specimens of human myocardium. *In vitro*, these cells multiply many times without losing their differentiation potential. However, explant CDCs are a complex mixture of heterogeneous cells; the proportion of c-kit^pos^ cells reported varies across different studies [13, 55]. Several research groups injected human explant CDCs into infarcted rodents and produced chimeric hearts with functional improvements [13, 30, 34, 55]. Johnston et al. also injected pig explant CDCs into infarcted pigs, resulting in injury improvement [33]. On the other hand, Shenje et al. reported that murine explant CDCs transplanted into mice with peri-infarcts produce chimeras without cardiomyocyte differentiation [56]. Andersen et al. prepared explant CDCs from neonatal rats and mice; however, these lacked cardiomyogenic potential on filtration [31]. Therefore, the mechanism by which the injected cells repair infarct damages remains controversial with the argument being between the following possibilities: (1) the injected cells stimulate the release of factors to help heal the damage; (2) they fuse with the host's ailing heart cells to replenish and compensate for the damaged mitochondria; or (3) the injected stem cells transform into heart muscle. Davis et al. point out that since the primary explants are a complex mixture of cells, subtle variations in the culture technique and conditions might alter the outcome [30]. Davis et al. later refined the technique by trying to limit phenotype drift during cell culture [35]. In this respect, Tang et al. isolated c-kit^pos^ cells from explant CDCs and preconditioned the cells prior to injection into animals with surgically induced myocardial damage [57]. They reported that hypoxic pretreatment of the cells results in a significant improvement in the recruitment of the injected cell to the ischemic myocardium.

## 4. Characteristics of Immunopurified c-Kit^pos^ Cardiac Stem Cells (CSCs)

Since the first report by Beltrami et al. [12], immunopurified c-kit^pos^ cardiac stem cells (CSCs) have been investigated for their self-renewing, clonogenic, and multipotent properties [15–20, 58–64]. c-Kit, also called KIT or CD117, is a cytokine receptor that binds to stem cell factor (SCF, also known as “steel factor” or “c-kit ligand”) [65]. c-Kit and its ligand (SCF) are known to be essential for hematopoiesis, melanogenesis, and fertility. Altered forms of this receptor may be associated with some types of cancer. Using transgenic mice with green fluorescent protein (GFP) under the transcriptional control of the c-kit locus, Tallini et al. recently observed c-kit^pos^ cells inside embryonic and postnatal developing hearts [66]; however, they decline rapidly in the initial weeks after birth [54]. These observations prove that c-kit^pos^ cells are involved at least in neonatal stages of cardiomyogenesis *in vivo*. Also, by damaging heart tissue in adult mice, c-kit^pos^ cells were observed transiently [54]. In human biopsies, 80% of cardiosphere-derived stem cells from the myocardial infarcted patients were c-kit and MDR-1 double positive [64]. The role of c-kit may change according to its expression level [22].

We studied the “stemness” and the cardiogenic potential of immunopurified c-kit^pos^ CSCs by splitting the purified cells in fractions and culturing them in bulk *in vitro* for over 40 passages. The long-term bulk culture lots were followed by mRNA marker analysis for “stemness” (Oct-4, Nanog, Klf-4, and Sox), cardiomyocyte lineage (GATA-4, MLC2v, cardiac actin, desmin, and connexin 43), adipocyte lineage (*α*PPAR), smooth muscle cells (smooth muscle actin), and skeletal muscle lineage (myogenin and desmin) gene expression. In the c-kit^pos^ CSCs bulk cultures from whole rat hearts, “stemness” and cardiac markers expressed at the onset of culture were lost in about half of the bulk cultures after 40 passages with more than half starting to express markers of differentiated cells [15], some even with double-lineage markers. This indicates that the phenotypes of c-kit^pos^ CSCs from whole adult hearts drift more toward differentiated cells during long-term culture [15]. The amount of GATA-4 mRNA expression drifts at different passages of the bulk culture lots [15]. However, when the source of c-kit^pos^ CSCs is limited to the left atrium, a higher proportion of bulk cultures exhibited markers for cardiac lineage and some produced beating cells when placed into differentiation media with cytokines Il-3 and SCF [18]. The bulk cultures were subjected to cloning by limited dilution methods, and the characteristics of clones obtained were not perfectly identical to the original bulk culture, perhaps due to heterogeneity within the bulk culture cells [15, 17, 18]. Nevertheless, the clones exhibit similar expression patterns as bulk cultures overall.

Among the bulk cultures, CSC-BC21 showed the greatest amount of spherical aggregate formation. A clone CSC-21E was derived from this bulk culture by limited dilution and maintained similar frequencies of spherical aggregate formation as the original bulk culture [15, 20]. CSC-21E cells showed two distinct morphologies, floating cardiospheres (flCS) composed of round cells and dish-attached cardiospheres (daCS) composed of flattened fibroblastic cells. These two different morphologies were obtained by switching plastic dishes; namely, flCS were obtained when cultured on bacterial dishes and daCS were obtained when transferred to cell culture dishes. The bacterial dishes were molded with virgin polystyrene to give a hydrophobic surface while the cell culture dishes were modified by plasma discharge to give a charged polystyrene surface. Trypsin treatment of daCS and transfer to bacterial dishes result in cells reverting to flCS, and this ability remained for at least 3 days after flCS were converted to daCS. Since this morphological change does not require any feeder cells or additional growth factors, clone CSC-21E appeared to be an attractive model for studying the effect of cell shape in determining cell properties.

## 5. Proteomic Comparison of Floating (flCS) and Attached (daCS) Cells of Clone CSC-21E

The two-dimensional (2D) fluorescence difference gel electrophoresis (DIGE) system uses two samples labeled separately with different fluorescent labels and is subjected to a 2D polyacrylamide gel electrophoresis after combining the two samples. The system can show overall protein profiles common to both samples and also is sensitive in detecting proteins that have different expression levels in the two samples. The clone CSC-21E was subjected to 2D DIGE to study the difference between protein expression levels in flCS and daCS. 

Overall protein profiles exhibited many “stemness” indicators observed in ESCs such as high levels of glycolytic and metabolic enzymes, a variety of chaperones or stress proteins, and annexins in both shapes [8–10, 20]. However, some “stemness” indicators including peroxiredoxin 1, T-complex protein 1, and translationally controlled tumor proteins were absent, suggesting that cardiospheres exist as an intermediately differentiated state with partial stem cell-like characteristics. Stress proteins are highly expressed in both flCS and daCS. Stress proteins are involved in regulating protein folding in various cell compartments and intracellular trafficking [67]. Some stress proteins are also implicated in the “stemness” of pluripotent cells as well as in the regulation of cell proliferation and differentiation [68–70].

**Figure 1:**
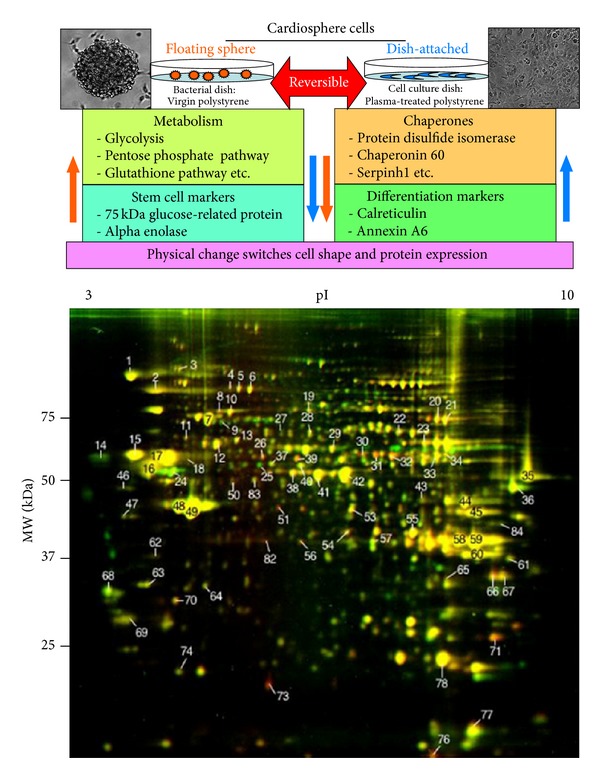
Upper Panel: Category of proteins differentially expressed in floating cardiosphere (flCS) cells and dish-attached cardiosphere (daCS) cells reported in Machida et al. [20]. Among the proteins successfully identified, about 44% were differentially expressed. Lower Panel: Comparative 2D-DIGE of CSC-21E whole cell lysates from flCS (red) and daCS (green) cultures. Proteins with similar amounts in flCS and daCS appear as yellow spots, whereas proteins differentially expressed show reddish (more abundant in flCS) or greenish (more abundant in daCS) spots. The figure was modified from Figure 3 of Machida et al. [20] with permission.

We also detected proteins which change expression levels in response to the shape change from floating aggregates to dish-attached expanded cells; see Figure 1. Metabolic enzymes such as glycolytic enzymes comprised about half of the proteins whose expression decreased upon cell attachment. In flCS cells, oxygen supply is limited by aggregation, which minimizes exposure to the environment. Levels of glutathione-S-transferase, pentose phosphate pathway enzymes, transaldolase, and a transaldolase isoform were higher in flCS than daCS. Furthermore, decrease in the levels of these proteins suggests that flCS can avoid oxidative stress better than daCS [7, 71]. In general, the defense system for oxidative stress is better in stem cells than differentiated cells [69, 70]. Therefore, since flCS have a superior antioxidative capacity compared to daCS, this also indicates that flCS cells maintain more “stemness” properties than daCS cells.

There are 2 other observations from the present CSC investigation that are worth noting. The first is the proteomic observation of a switch in chaperons that occurs when floating cardiosphere cells become dish-attached cells. Among stress proteins, Hsp90, chaperonin 60, and calreticulin exhibit increased expression in attached cells. Hsp90 is known to participate in the stabilization and refolding of denatured proteins after stress and also plays a key role in the maturation of signal transduction proteins and assembly of myofibril filaments [72, 73]. Calreticulin is the most notable among the stress proteins upregulated upon cell attachment, because it is known to be essential for cardiac development [74]. The increase in calreticulin expression induces increase in the levels of both vinculin and N-cadherin, which play an important role in increasing cell adhesiveness to their substratum and communication with their neighboring cells. The upregulation of Hsp90 and calreticulin after the transition from flCS to daCS clearly indicates that this is a key step toward forming cardiomyocytes.

Notably, we found high levels of annexin family proteins in CS cells. Annexins are a family of membrane-binding proteins, and they bind to phospholipid surfaces in a Ca^++^-dependent manner. Annexins A1, A2, and A6 are also actin-binding proteins [75]. We observed high expression levels of annexins A1 and A2 and distinct expression of annexins A4 and A5 in both flCS and daCS. On the other hand, annexin A7 expressed in flCS cells was decreased in daCS while the expression of annexin A6 was increased in daCS. The change from annexin A7 to A6 concomitant with flCS/daCS switching might suggest a different requirement for the cytoskeletal organization and formation of the plasma membrane in these cells. Molecular chaperones help many signaling molecules maintain their activation-competent states and regulate various signaling processes [76]. Further investigation in this area may aid the development of chaperone-modulating chemicals and drugs that might maintain the “stemness” or manipulate the differentiation of certain cells. 

The second is the control of cell fate by physical interactions with the substrate surface. Guilak et al. elegantly reviewed the role of the substrate extracellular matrix (ECM) in inducing changes in cell shape [77]. Interestingly, the artificial alteration of physical structure of ECM resulted in cell shape from rounded to flattened morphology and consequently influenced cell fate. Cell adhesion to ECM results in activation of Rho, a member of the Ras superfamily of small GTP-binding proteins, which is involved in signaling a switch between adipogenesis and myogenesis [78, 79]. The switch between floating and adherent cells alters not only the morphology of the cell, but also the expression of functional proteins. Since the present experimental system does not involve ECM for cell adhesion, it may be instrumental for exploring how mechanosensitive components of the cell surface trigger molecules that determine cell fate.

## 6. Stem Cell Niche and Cardiospheres

In 1978, Schofield proposed the “niche” hypothesis to describe the physiologically limited microenvironment that supports stem cells [80]. Since then, stem cells are thought to exist in specialized microenvironments known as niches, which play an important role in microenvironment-stem cell interactions [81]. Stem cell niches are often located in regions of low oxygen supply [82] and require elevated glycolysis. Kondoh et al. reported that the proliferative capacity of murine ES cells correlates with high glycolytic enzyme activity and low oxygen consumption [83]. Unwin and Whetton identified several components of anaerobic glycolysis that exhibit decreased expression upon the loss of stem cell characteristics [7]. 

A primary function of the niche is to anchor stem cells to the microenvironment suitable in maintaining their healthy progenitor properties. To stabilize stem cell characteristics during long-term culture, it is critical to understand the nature and stimulation of the stem cell niche. The proteomic profile of floating CSs indicates that cardiospheres are adapted for hypoxia. The low oxygen pressure in stem cell niches was recently identified [84, 85]. Although we inhale an ambient oxygen tension of 21% (160 mmHg), the partial pressure of oxygen (pO_2_) in organs and tissues inside our body drops to 2–9% (14–65 mmHg), which is considered normal for cells [86, 87]. Consequently, in the routine laboratory-scale cell culture, the ambient pO_2_ of 21% is too high for cells, and the benefits of adjusting to their physiological pO_2_ are obvious and require serious consideration. Toussaint et al. have cited many examples of the physiological pO_2_ level of 2–9% providing benefits to the maintenance of a variety of cells [85]. Slightly lower oxygen tensions are indicated for several stem cell niches [84, 85]. Low O_2_ tension prevents spontaneous differentiation of human embryonic stem cells [88] and is required to activate HIF, which is a master gene regulator that controls several dozen genes in response to hypoxia [89, 90].

## 7. Proteomic Comparison Studies in Spheroids

 Spheroid-like structures were formed in various stem cells and cancer stem cells such as neurospheres, dental follicle precursor cells, and cancer cells [91–94]. Proteomic comparisons were performed between monolayer and tumor spheroids, and it was suggested that cell-stress proteins, such as HSP-90, 70, and 60 were upregulated in spheres [92, 93]. Moreover, in prostate cancer stem cells (DU145), CD44^+^ spherical forms maintained stemness, suggesting that the spheroid state may be advantageous for the maintenance of stem cells [94]. The metastatic activity of these cells is regulated by the TGF-beta signaling pathway [93]. Furthermore, the studies using EBs suggest that heat shock proteins (HSPA5 and HSPA8) are upregulated in undifferentiated states, suggesting that alteration of expression levels of stress proteins is associated with changes by differentiation stages [95].

## 8. Conclusion

Proteomic analysis of various spheroid-like cell aggregates reveals that they have distinct protein profiles common among them, that is, upregulation of certain metabolic enzymes adaptable to low oxygen condition and the expression level of some stress proteins when compared with monolayer cultures. Next questions are to unravel the relationship between the factors involved in cell shape, surface mechanosensitivity, and oxygen pressure and between the alteration in protein profiles. Understanding the regulation in protein profile changes is an area expected to provide further insights. Thus technology to manipulate the protein profile may lead to the development of regenerative therapy.
